# Isopycnal mixing of interhemispheric intermediate waters by subthermocline eddies east of the Philippines

**DOI:** 10.1038/s41598-019-39596-2

**Published:** 2019-02-27

**Authors:** Feng Nan, Fei Yu, Qiang Ren, Chuanjie Wei, Yansong Liu, Shuhui Sun

**Affiliations:** 1 CAS Key Laboratory of Ocean Circulation and Wave Studies, Institute of Oceanology, Chinese Academy of Sciences, Qingdao, China; 2 Pilot National Laboratory for Marine Science and Technology (Qingdao), Qingdao, China; 3 Center for Ocean Mega-Science, Chinese Academy of Sciences, Qingdao, China; 4 College of Earth Science, University of Chinese Academy of Sciences, Beijing, China

## Abstract

Both sporadic observations and modelling studies suggest that subthermocline eddies (SEs) exist east of the Philippines, where interhemispheric waters meet. However, effects of SEs on water mass mixing have never been observed. Here, using data from mooring and buoy deployed in the frontal region of the interhemispheric water masses, we show for the first time that the SEs act as an “underwater mixer” of intermediate waters from north and south Pacific oceans. The SEs have typical swirl speeds of 0.1~0.4 m s^−1^ between 200 and 800 m depth with a dominant period of ~90 days. Variation in intermediate water salinity also had a period of ~90 days, lagging eddy speed by ~8 days. Horizontal eddy diffusivity representative of eddy mixing rate was quantified using a mixing-length framework. Horizontal eddy diffusivity had both surface and subthermocline maxima. The vertically varying eddy diffusivity can be used to improve parameterization of eddy stirring in the tropical Pacific by coarse-resolution ocean climate models. The effect of the SEs on mixing of intermediate water masses seems not resolved by available eddy-resolving ocean models typically used for this region.

## Introduction

Mesoscale eddies are energetically dominant and ubiquitous in the world oceans^1,2^, playing important roles in ocean circulation, heat and salt transport, mixing and redistribution of water masses, *etc*.^3–7^ Mesoscale eddies can be classified as surface and subsurface eddies based on the vertical structure of isopycnals^8^. The former are characterized by either upward or downward isopycnals, and have been extensively studied over the past decades using remote sensing data^1,2^. In recent years, subsurface eddies have been characterized as isopycnals with a lens-type structure^8,9^, such as intrathermocline eddies^10–13^, mode-water eddies^14,15^, and subthermocline eddies (SEs)^16–22^. Vertical structure of subsurface eddies cannot’ be detected remotely, but rather rely on *in situ* measurements. Thus, the underlying dynamics of subsurface eddies remain elusive.

Modelling results show that there are two groups of SEs off the Philippine coast leading to higher eddy kinetic energy in the subthermocline (>26 σ_*θ*_)^20,21^. One originates from the southeast, and the other originates from the east. The two groups of SEs have similar radius of ~150 km and propagation speed of ~0.12 m s^−1^. Their cores are located between 200 and 800 m depth. Both barotropic and baroclinic instabilities contribute to SE formation^21,22^. Subthermocline currents (*e.g*., North Equatorial Undercurrent (NEUC) and Mindanao Undercurrent (MUC)) in this region are dominated by intraseasonal variability of 70–120 day, which are suggested to be modulated by SEs^23,24^. However, information about SEs in this region is still limited because of a lack of long-term observations.

**Figure 1:**
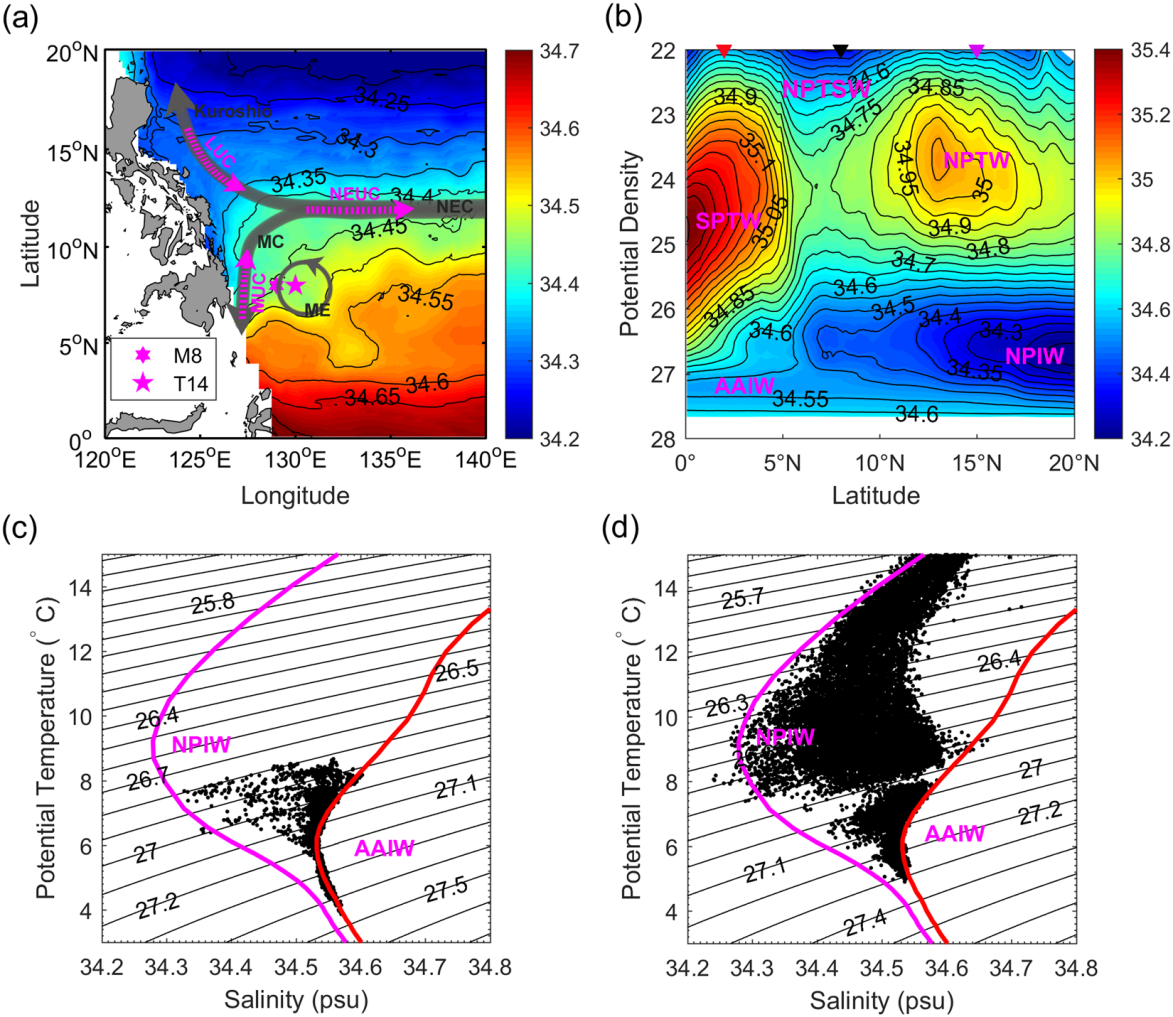
Water mass characteristics east of the Philippines. (**a**) Climatological salinity (psu) distribution at 26.8 *σ*_*θ*_ derived from gridded Argo data. Moorings M8 and buoy T14 are located at 129°E and 130°E, 8°N, respectively. Schematic of major currents are indicated. NEC, MC, ME, MUC, NEUC, and LUC stand for North Equatorial Current, Mindanao Current, Mindanao Eddy, Mindanao Undercurrent, North Equatorial Undercurrent, and Luzon Undercurrent, respectively. (**b**) Climatological salinity (psu) distribution along 130°E from gridded Argo data. The red and purple triangles represent the associated location of temperature and salinity profiles in (**c**,**d**), while the black triangle is near the site of buoy T14. (**c**,**d**) Scatterplots of potential temperature (*θ*) against salinity (*S*) measured by mooring M8 (**c**) and buoy T14 (**d**). Isolines of neutral density (kg m^−3^) are given by the black curves in (**c**,**d**).

The tropical northwestern Pacific is a region where water masses converge^25^ (Fig. 1). Interhemispheric waters that meet here are transferred by the Western Boundary Currents^26,27^. Along the Philippine coast, the Mindanao Current (MC) is formed by the bifurcation of the westward North Equatorial Current (NEC) and flows equatorward carrying the North Pacific tropical water (NPTW) and the North Pacific Intermediate Water (NPIW)^28^. In the subthermocline (>26 σ_*θ*_), the MUC transports the Antarctic intermediate water (AAIW) poleward (Fig. 1a)^29,30^. Semiannual variability of MC and MUC is dominant and can contribute to interhemispheric intermediate water masses exchange^31^. Off the Philippine coast, both sporadic *in situ* observations and modelling studies suggest the existence of SEs with dominant intraseasonal variability^18–22^. It is known that eddies can mix water masses and influence the distribution of tracers such as heat and salt^32,33^. The effect of SEs on isopycnal mixing of interhemispheric intermediate waters in this region remains unclear.

Understanding mixing by mesoscale eddies is of both theoretical interest and practical importance^34^. In this study, based on data from mooring and buoy deployed in the frontal region of interhemispheric water masses, we show the characteristics of the SEs and their effects on isopycnal mixing of intermediate waters from north and south Pacific oceans. Horizontal eddy mixing length and diffusivity are quantified using a mixing-length framework based on observations, which can be used to improve parameterization of eddy stirring in this region for coarse-resolution ocean climate models.

## Data and Methods

### Mooring and buoy measurements

To investigate water mass mixing, a subsurface mooring system (M8) was deployed in the frontal region of interhemispheric water masses at 129°E, 8°N (Fig. 1a) from January 2016 to April 2017. Similar to earlier studies^24,29^, two (one upward looking and one downward looking) 75 kHz Acoustic Doppler Current Profilers (ADCPs) manufactured by Teledyne RD Instruments were installed on the main float of M8 at 400 m water depth to measure current velocity in the upper 800 m. The ADCPs were configured to measure hourly with a standard bin size of 8 m. A 1-day running mean was applied to the raw ADCP velocity data to remove tidal and synoptic signals and linearly interpolated at 8-m intervals profiles^31^. Six conductivity-temperature-depths sensors (CTDs) manufactured by Sea-Bird Scientific were installed at depths of 350, 400, 500, 600, 700, and 800 m. These CTDs were configured to measure temperature and salinity every 10 minutes, and a 1-day running mean was applied to the data.

The buoy (T14) was used to obtain temperature and salinity data from surface to 750 m. Buoy T14 is part of the Triangle Trans-Ocean Buoy Network (TRITON) that is located at 130°E, 8°N (Fig. 1a). The TRITON buoys were deployed since 1998 by the Japan Agency for Marine-Earth Science and Technology (JAMSTEC) as part of the Tropical Ocean Climate Study (TOCS)^35^. The CTDs were installed at 1.5, 25, 50, 75, 100, 125, 150, 200, 250, 500, and 750 m depth. Buoy T14 provides daily temperature and salinity data from June 2003 to March 2013, with data gaps in 2006 and 2007. Thus, for this study, we used data obtained from 2008 to 2012.

### Estimation of mixing length and horizontal diffusivity

Horizontal eddy mixing rate can be quantified through eddy diffusivity (*κ*_*h*_)^36^. Until now, research on the spatial and temporal distribution of eddy diffusivity has been limited. The mixing length (*λ*) represents the distance a fluid parcel is transported before significant irreversible mixing occurs^32^. A mixing-length framework that relates observed salinity anomalies on isopycnal surfaces to gradients of the mean salinity field is used to estimate horizontal *λ* and *κ*_*h*_ as follows^32,33^:

1 $$\lambda ={\langle S^{\prime} S^{\prime} \rangle }^{\frac{1}{2}}/\langle |\nabla \{S\}|\rangle $$

2 $${\kappa }_{h}={{\rm{c}}}_{0}\lambda {u}_{rms}$$

where *S*′ is the isopycnic salinity anomaly, ${u}_{rms}={\langle u{^{\prime} }^{2}+v{^{\prime} }^{2}\rangle }^{1/2}$ is the root-mean-square eddy velocity, *c*_0_ is the constant mixing efficient (0.16), braces {} indicate a one-year running mean, and brackets 〈〉 are for the temporal average over all years^32^. *u′* and *v′* are zonal and meridional velocity anomalies, respectively. Mean salinity gradient $\langle |\nabla \{S\}|\rangle$ was calculated using the gridded Argo data set, *i.e*., Grid Point Value of the Monthly Objective Analysis (MOAA GPV). The MOAA GPV data is a global 1° × 1° dataset of monthly temperature and salinity starting from January 2001 to the present^37^. In this study, we used data obtained from 2008 to 2012 to correspond with data measured by buoy T14.

### Model data

To test whether the available eddy-resolving ocean models can reproduce the effects of the SEs on intermediate waters mixing, the OFES (Oceanic General Circulation Model for the Earth Simulator) model data without data assimilation and the HYCOM (Hybrid Coordinate Ocean Model) model data with data assimilation were selected. The OFES (HYCOM) model has a horizontal resolution of 0.1° × 0.1° (1/12° × 1/12°) and a total of 54 (32) levels. These two eddy-resolving models have been widely used in a number of earlier studies to investigate currents, eddies, and water masses in the northwestern Pacific Ocean^20,21,23,27–29,38^. Detailed descriptions of the models are provided by those studies. In this study, daily (3-day intervals) temperature and salinity data are used from the HYCOM (OFES) output from 2008 to 2012.

## Results

### Isopycnal mixing of intermediate waters

Off the Philippine coast is a region of mixing of interhemispheric water masses. Water masses originating from the northern and southern hemispheres intersect here and are transformed through isopycnal/diapycnal mixing. In this study, we focus on isopycnal water mixing with *κ*_*h*_~*O*(10^3^) m^2^ s^−1^, which is mainly induced by eddies and significantly greater than diapycnal mixing with diapycnal diffusivity *κ*_*ρ*_~*O*(10^−6^) m^2^ s^−1^ in this region^39^. Different water masses were often identified by salinity extremes^27,40^. Climatological salinity distribution along 130°E from gridded MOAA GPV data is shown in Fig. 1b. Low salinity water in the upper mixed layer characterizes the North Pacific Tropical Surface Water (NPTSW) that is formed in the northwestern Pacific warm/fresh pool where precipitation exceeds evaporation. In the thermocline (<26 σ_*θ*_) and below the NPTSW, two subsurface salinity maxima exist. The southern one is the South Pacific tropical water (SPTW), with maximum salinity of ~35.4. It is saltier than the northern one, called NPTW, with maximum salinity of ~35.2. Both the NPTW and SPTW are formed in the middle of the Pacific subtropical gyres where evaporation is far higher than precipitation^40^. In the subthermocline (>26 σ_*θ*_), there exist two subsurface salinity minima. The southern one, named the AAIW, has a minimum salinity of ~34.6 and is saltier than the northern one, called the NPIW with minimum salinity of ~34.2. Note that the depths of the SPTW and the AAIW in the southern hemisphere are greater than those of the NPTW and NPIW in the northern hemisphere.

The MC is strongest near the Philippine coast and can extend approximately 250 km offshore (128.75°E)^28^. The MUC is 50–80 km off the Philippine coast. Meridional MC and MUC have significant semiannual variability and play an important role in interhemispheric intermediate water exchange^29^. Most moorings have been deployed at ~127°E near the Philippine coast to observe variability of the MC and MUC^23,28,29,31^. Here, to observe eddy effects on water masses mixing, a subsurface mooring was deployed at 129°E, 8°N off the Philippine coast, where the eddy activity is dominant^20–22^. It is also in the frontal region of the interhemispheric water masses (Fig. 1a,b).

Salinity is a good indicator for investigation of intermediate water mixing^31–33^. Figure 1c shows a scatterplot of *θ*-*S* measured from six CTDs deployed on mooring M8. The magenta and red curves show representative *θ*-*S* diagrams at 15°N and 2°N, respectively. The minimum salinity of the AAIW is ~34.55 at 2°N at ~27.2 σ_*θ*_, while the minimum salinity of the NPIW is ~34.3 at 15°N at 26.6 σ_*θ*_. The most striking feature of Fig. 1c is that the observed salinity of intermediate water at 8°N is almost between salinities of AAIW and NPIW, which reflects the strong mixing of these two water masses. Data are not available above 350 m, and so water mass characteristics above 26.8 σ_*θ*_ are not shown. There was a nearby TRITON buoy (T14) at 130°E, 8°E. Figure 1d shows a scatterplot of *θ*-*S* measured by 11 CTDs deployed on buoy T14. The results suggest that the AAIW and the NPIW are sufficiently mixed between 26.3 and 27.1 σ_*θ*_ off the Philippine coast.

### Observed SEs off the Philippine coast

**Figure 2:**
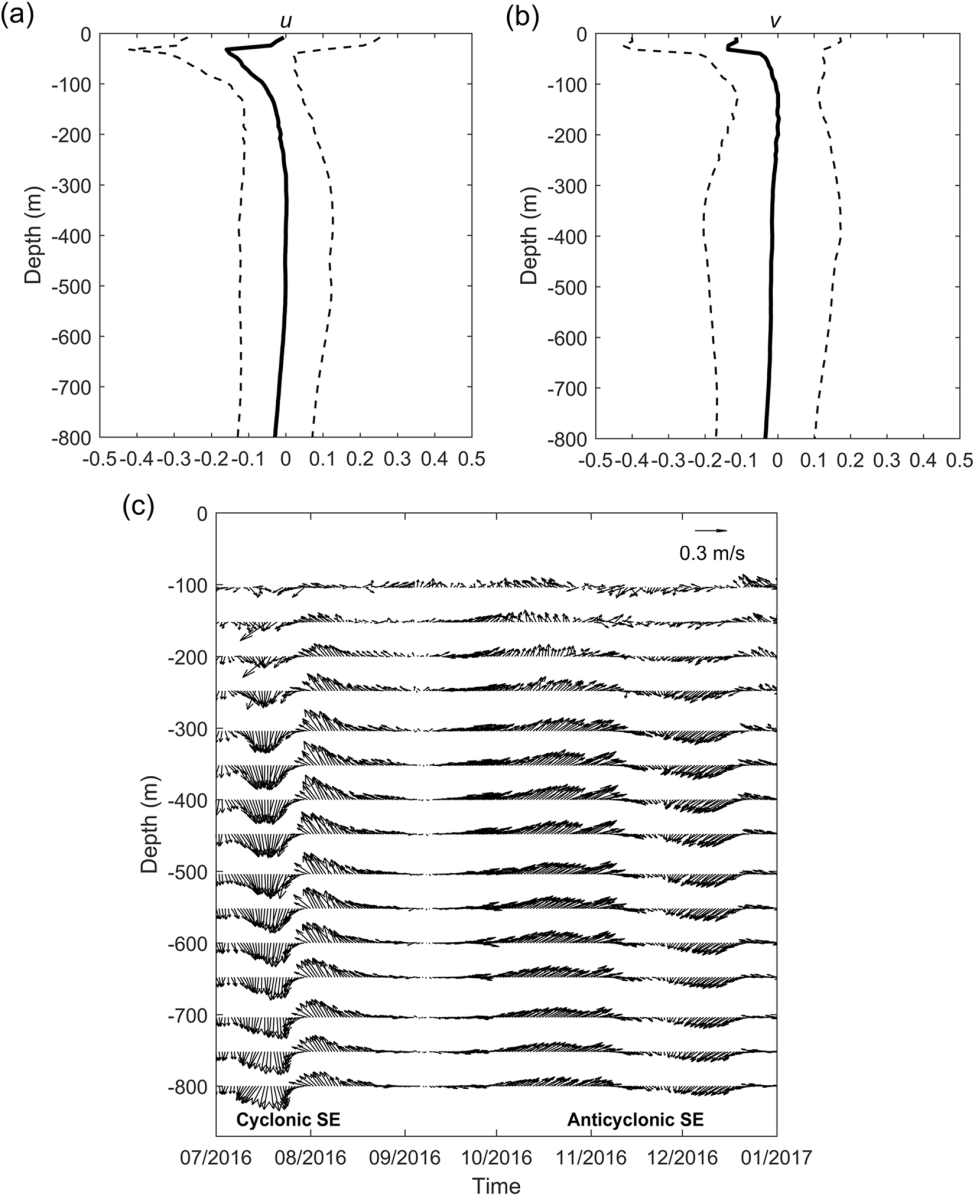
Measurements of velocity (m/s) above 800 m by mooring M8. Mean (**a**) zonal (*u*) and (**b**) meridional (*v*) velocity (black solid lines) and its standard deviations (dotted lines). (**c**) Daily velocity variations during July-December 2016 showing vertical structures of a cyclonic SE and an anticylonic SE.

**Figure 3:**
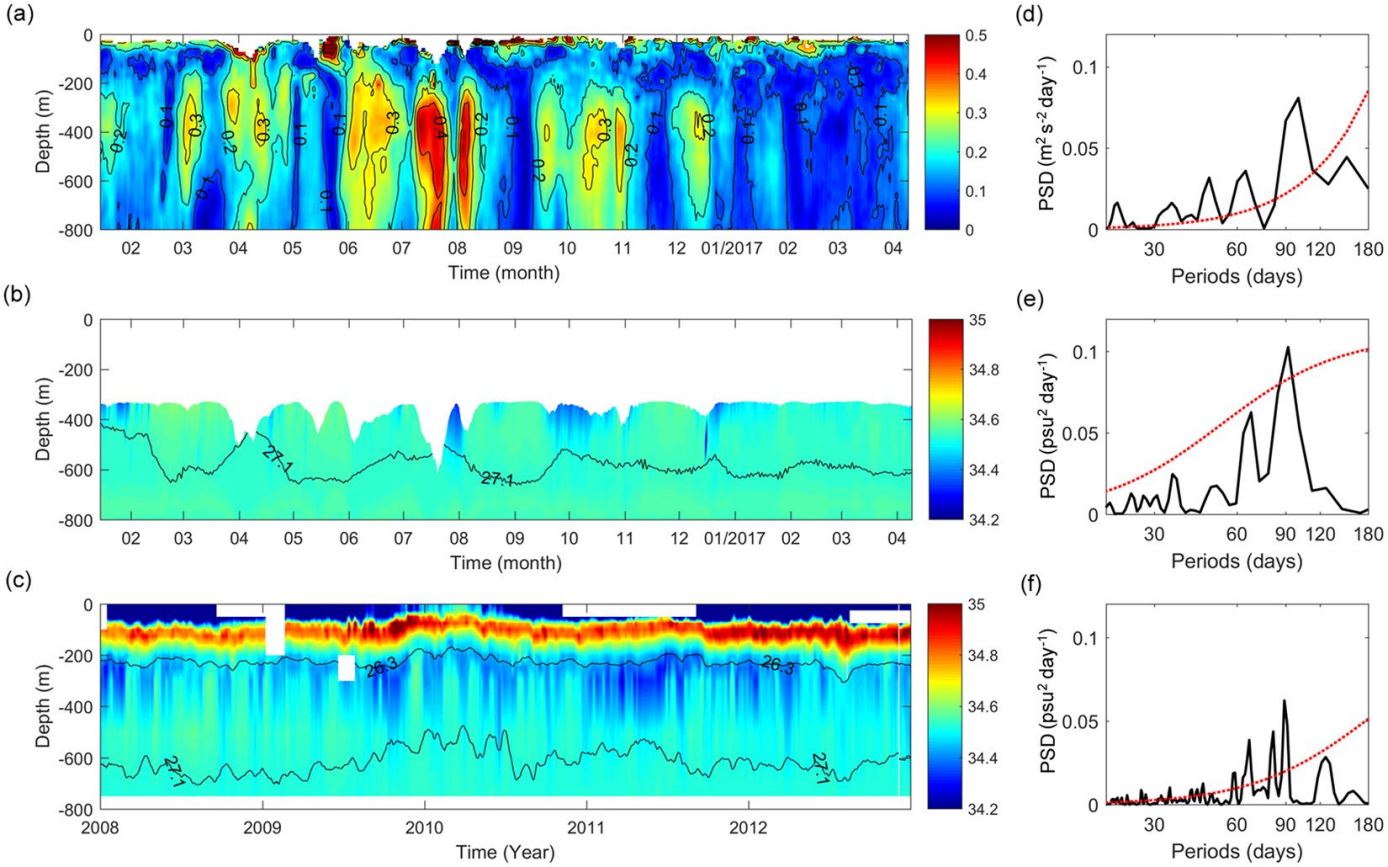
Measurements of eddy velocity magnitude (**u**′) and salinity. (**a**) Daily **u*′*** (m s^−1^) measured by mooring M8 sampled from January 2016 to April 2017. (**b**) Daily salinity (psu) measured by mooring M8 overlaid with 27.1 *σ*_*θ*_ in black contour. (**c**) Daily salinity (psu) measured by buoy T14 sampled during 2008–2012 overlaid with 26.3 and 27.1 *σ*_*θ*_ in black contours. (**d**) Power spectral density (PSD) for daily **u′** (**a**) averaged between 200 and 600 m. (**e**) PSD for daily salinity (**b**) averaged between 26.3 and 27.1 *σ*_*θ*_. (**f**) PSD for daily salinity (**c**) averaged between 26.3 and 27.1 *σ*_*θ*_. The red dashed lines in (**d**–**f**) denote the 95% significance level.

From Fig. 2, the mean current measured by mooring M8 is south-westward with maximum velocity of ~0.15 m/s above 200 m. This is caused by cyclonic currents of the Mindanao Eddy (ME), since the mooring M8 is deployed in the northwestern of the ME based on satellite altimeter data (Figure not shown). The mean current below 200 m is near zero. Interestingly, the standard deviations of both zonal and meridional currents are larger than the mean currents, indicating that eddy activity is predominant. The maximum standard deviations of both zonal and meridional currents reach 0.25 m/s above 200 m. This is because the ME is a recirculation of the MC rather than a quasi-stationary eddy confined above the thermocline^38,41^. Below 200 m, the maximum standard deviation appears at ~400 m where the maximum speed of the SEs appears (Fig. 3a). The standard deviation of the zonal current is approximately 0.1 m/s, which is a little smaller than that of the meridional current (0.15 m/s). Figure 2c shows prototypical vertical structures of a cyclonic SE and an anticylonic SE. It can be seen that they appeared below 200 m with maximum velocity larger than 0.3 m/s.

Variations of SE activities are shown clearly in Fig. 3a. Daily eddy velocity magnitude (**u′** = (*u′* ^2^ + *v′* ^2^)^1/2^) was measured by mooring M8 from January 2016 to April 2017. It can be seen that the SEs are located between 200 and 800 m as identified by velocity contours greater than 0.1 m s^−1^. The maximum swirl speed reaches 0.4 m s^−1^ at ~400 m depth. Previous modelling results show that the dominant time scale of the SEs range between 50 and 80 days^20,21^. However, power spectrum for daily **u′** averaged between 200 and 600 m shows that the SE in this region exhibits intraseasonal variability with periods between 50 and 100 days. The power is greatest at 90 days. This result provides observational evidence for intraseasonal variability of the SEs in this region.

### Mixing effect of the SEs

**Figure 4:**
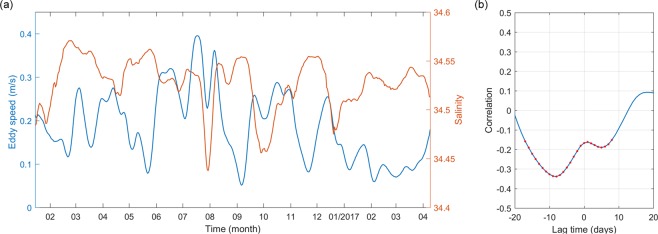
Correlation between **u**′ and salinity. (**a**) Daily **u**′ (m s^−1^) averaged between 26.3 and 27.1 *σ*_*θ*_ and daily salinity (psu) averaged between 200 and 600 m measured by mooring M8. (**b**) Lead-lag correlation between **u**′ and salinity in (**a**). Negative lag time indicates **u**′ lagging salinity. The red dots denote correlation above the 99% significance level.

It is known that mesoscale eddies can stir salinity along density surfaces (spice variance), playing an important role in the mixing of water masses^30,31^. Figures 3b,c show daily salinity measured by mooring M8 from January 2016 to April 2017 and buoy T14 from 2008 to 2012, respectively. Saltier and fresher waters alternately appear in the intermediate layers between 26.3 and 27.1 σ_*θ*_. Variation in intermediate waters as indicated by salinity variation averaged between 26.3 and 27.1 σ_*θ*_ also have a dominant period of 90 days (Fig. 3e,f), which is consistent with occurrence frequency of the SEs. Variations of intermediate water and the eddy current as measured by mooring M8 seem opposite (Fig. 4a). The correlation coefficient between eddy speed and salinity is greatest (*r* = −0.32, exceeding 99% significance level) when salinity lags eddy speed by 8 days (Fig. 4b). The 8-day lag reflects the response time of local salinity to the eddy. Thus, we can conclude that intermediate water mixing off the Philippine coast observed by mooring M8 and buoy T14 is caused by water mass mixing from the SEs. The SEs seem to act as an “underwater mixer” of interhemispheric intermediate water masses.

**Figure 5:**
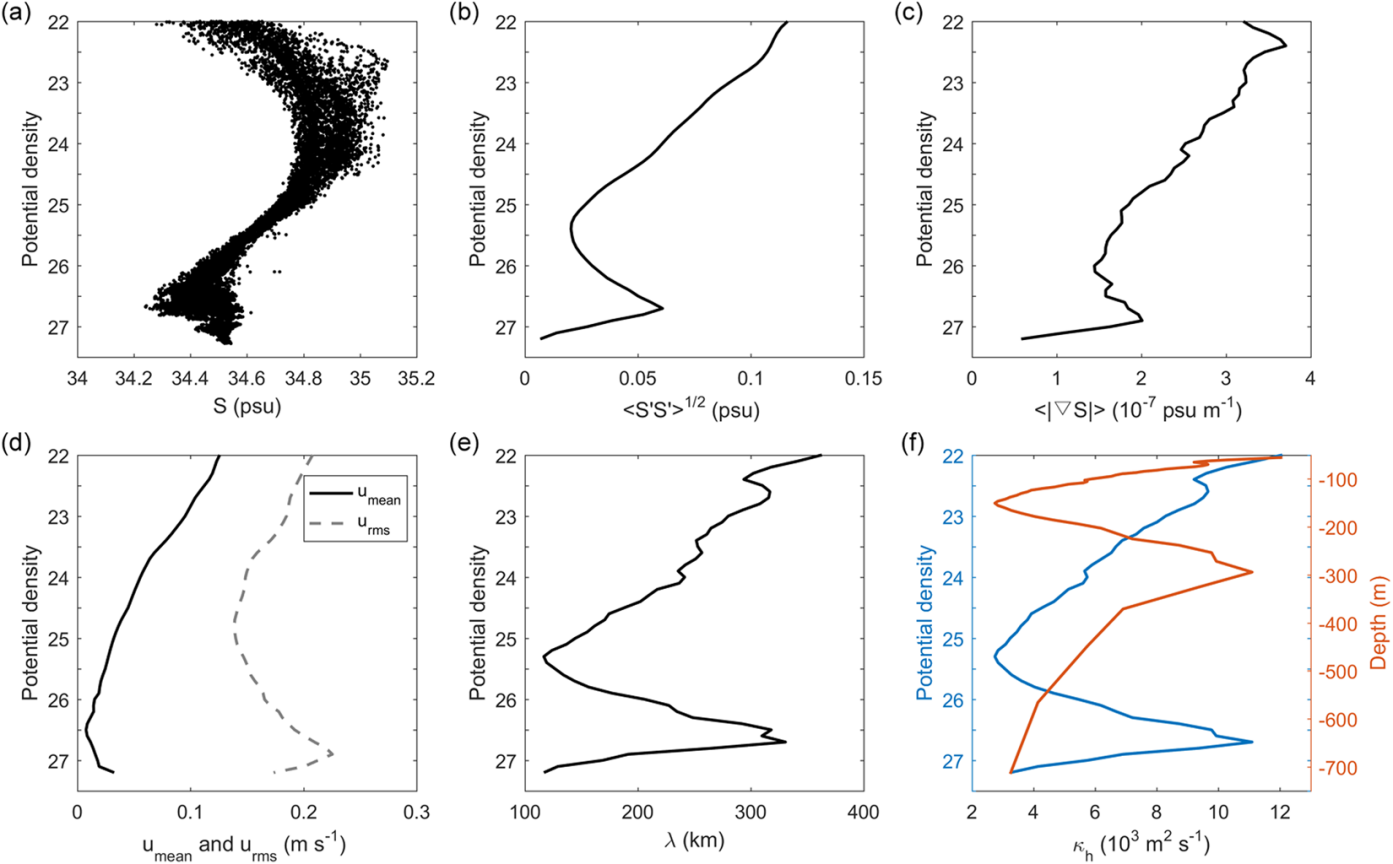
Vertical distributions of estimated eddy mixing length (*λ*) and diffusivity (*κ*_*h*_) as a function of potential density. (**a**) Salinity (psu) measured by buoy T14. (**b**) Salinity standard deviation (psu) in (**a**). (**c**) Horizontal gradient of mean salinity (psu m^−1^) derived from the monthly Argo dataset. (**d**) Mean velocity magnitude (m s^−1^) and its standard deviation measured by mooring M8. (**e**) Estimated *λ* (km) based on Equation (1). (**f**) Estimated *κ*_*h*_ (m^2^ s^−1^) based on Equation (2). It is also given as a function of depth (m).

Eddy stirring effects cannot be resolved by most coarse-resolution ocean climate models, and instead have to be parameterized. The value for parametrization of eddy diffusivity (*κ*_*h*_) is typically of 1000 m^2^ s^−1 ^^42,43^, which sometimes is higher in the upper ocean and lower below the thermocline^44^. Thus, quantification of vertical variation of eddy diffusivity will improve parameterization of mixing in coarse-resolution models. Based on observed variation in salinity by buoy 14 and eddy activities by mooring M8, horizontal eddy mixing length (*λ*) and diffusivity (*κ*_*h*_) are estimated using Equations (1) and (2), respectively (Fig. 5). According to Fig. 5a–d, salinity variance, gradient, and current velocity are largest in the surface layer and decrease with depth. There exists a subthermocline maximum in the intermediate layer at ~26.8 σ_*θ*_. Figure 5e,f show the vertical distributions of estimated *λ* and *κ*_*h*_ as a function of potential density and depth. *λ* and *κ*_*h*_ are not uniform but vary vertically. They both have surface and subthermocline (at ~26.8 σ_*θ*_) maximum. *λ* varies from 120 to 350 km, which is consistent with the eddy diameter in this region^22^. *κ*_*h*_ varies with depth by about an order of magnitude from 3 × 10^3^ m^2^ s^−1^ to 1.2 × 10^4^ m^2^ s^−1^. The averaged *κ*_*h*_ is 6.4 × 10^3^ m^2^ s^−1^, and the subthermocline maximum *κ*_*h*_ is 1.1 × 10^4^ m^2^ s^−1^. The results based on observations can be used to improve parameterization of eddy stirring in this region for ocean climate models.

## Discussion

In this study, based on data from mooring and buoy deployed in the frontal region of the interhemispheric water masses, we show for the first time that SEs act as an “underwater mixer” of isopycnal mixing of North Pacific and South Pacific intermediate waters off the Philippine coast. The SEs have typical swirl speeds of 0.1–0.4 m s^−1^ between 200 and 800 m depth and dominant intraseasonal variability. Horizontal eddy mixing length and diffusivity are estimated using a mixing-length framework. Maximum eddy mixing length and diffusivity appear at the surface, decrease with depth, and exhibit a subthermocline maximum. Ocean models for climate studies are still too coarse to resolve mesoscale eddies and have to be parameterized^36,42–44^. Arguably, ocean models are limited by observations of eddy diffusivity with spatial and temporal variations^36^. With more and more subthermocline/intrathermocline/mode-water/subsurface eddies being observed around the global ocean^45–47^, the typical value of eddy diffusivity (1000 m^2^ s^−1^) which is higher in the upper ocean and lower below the thermocline is being questioned. Significant deviation from the mean eddy diffusivity in the subsurface will induce more mixing, a process missed in oceanic models. The results presented here that are based on observations can be used to improve parameterization of eddy mixing in this region for climate and ocean models.

**Figure 6:**
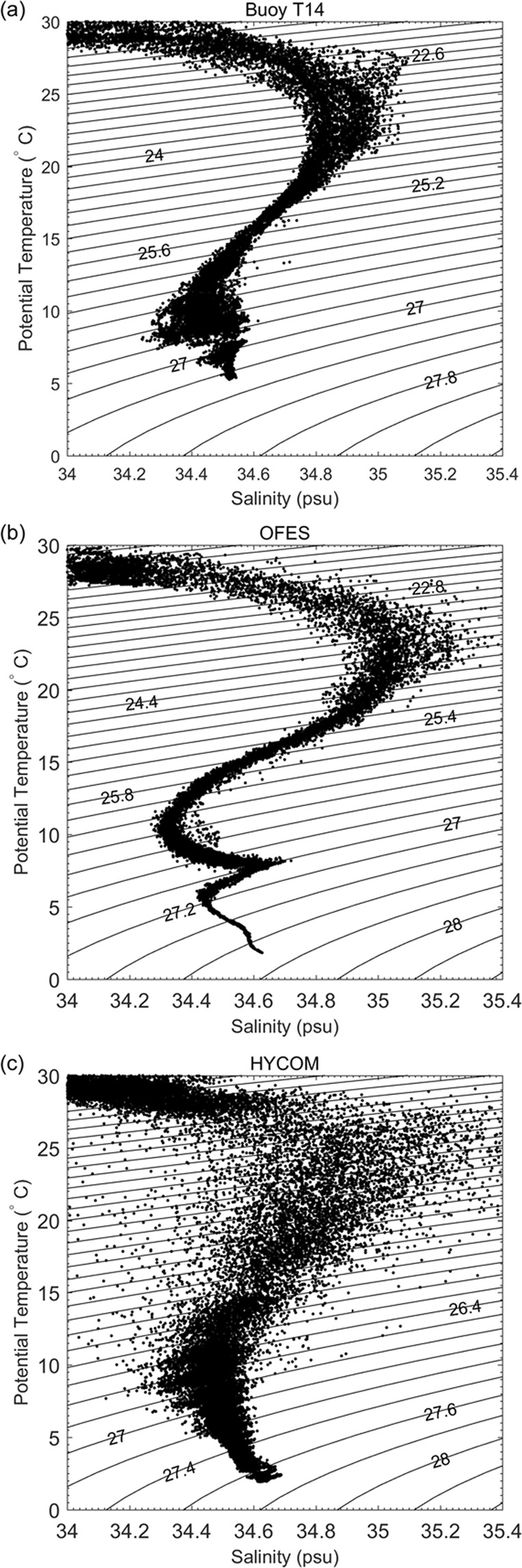
Scatterplots of *θ*-*S* at 130°E, 8°N from 2008 to 2012. (**a**) Daily temperature and salinity data are from buoy T14. (**b**) Temperature and salinity data with 3 days interval are from OFES output without data assimilation. (**c**) Daily temperature and salinity data are from HYCOM output with data assimilation.

**Figure 7:**
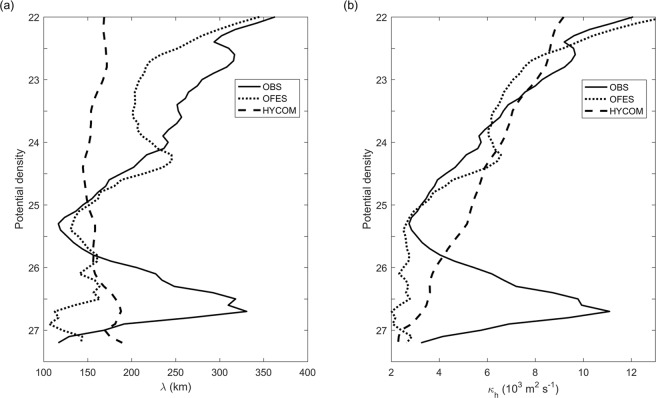
Vertical distributions of *λ* (**a**) and *κ*_*h*_ (**b**) as a function of potential density at 130°E, 8°N derived from observations (see Fig. 4e,f), OFES and HYCOM outputs during 2008–2012.

Mesocale eddies can be resolved in high-resolution ocean models. The eddy-resolving OFES and HYCOM model outputs have been extensively analyzed by a number of earlier studies to investigate ocean circulation and eddies in the northwestern Pacific Ocean^20,21,23,27–29,38^. However, the effect of SEs on intermediate waters mixing is not reproducible by these eddy-resolving ocean models. According to Fig. 6, the OFES model without data assimilation reproduces water mass structure within the thermocline. However, in the subthermocline, the AAIW and the NPIW are not mixed, which is not in accordance with observations presented here. The result of the HYCOM model with data assimilation is even less accurate. The salinity varies larger than the observed results. Meanwhile, the subthermocline maximum is not clear. According to Equations (1) and (2), vertical distributions of *λ* and *κ*_*h*_ at 130°E, 8°N were derived from OFES and HYCOM outputs during 2008–2012 (Fig. 7). It can be seen that eddy mixing length and diffusivity are largest in the surface layer and decrease with depth. Compared with observational results, there are not subthermocline maxima in the intermediate layer at ~26.8 *σ*_*θ*_ for the two models. The poor representation of eddy-mixing effects is the major cause of these biases. It is recommended that these two models should be applied with caution when investigating water mass mixing and transformation in the tropical Pacific Ocean.

In addition, this study focuses on the effects of eddies on isopycnal water mixing since horizontal mixing is more influential than vertical mixing in determining meso- to large-scale salinity distribution^44^. It has been observed that the ME can enhance diapycnal mixing at its flanks. The diapycnal diffusivity (*κ*_*ρ*_) is elevated by an order of magnitude due to eddy-induced geostrophic shear^39^. The SEs off the Philippine coast can also induce strong vertical current shear in the subthermocline (Fig. 2b), which is suggested to enhance diapycnal mixing. Carefully designed observations may provide more insights on the effects of SEs on diapycnal mixing.

## Data Availability

Buoy T14 data can be downloaded from the TRITON Web site http://www.jamstec.go.jp/jamstec/TRITON/real_time/. The MOAA GPV data are publicly available at http://www.jamstec.go.jp/ARGO/argo_web/argo/?lang=en. The OFES data and the HYCOM data are publicly available at http://apdrc.soest.hawaii.edu/data/ and https://hycom.org/, respectively. The mooring M8 data that support the findings of this study are available from the corresponding author upon request.
